# Motion Capture Technologies for Athletic Performance Enhancement and Injury Risk Assessment: A Review for Multi-Sport Organizations

**DOI:** 10.3390/s25144384

**Published:** 2025-07-13

**Authors:** Bahman Adlou, Christopher Wilburn, Wendi Weimar

**Affiliations:** Sport Biomechanics Lab, School of Kinesiology, Auburn University, 301 Wire Rd, Auburn, AL 36830, USA

**Keywords:** biomechanics, MoCap, sports science, wearable sensors, ecological validity, validation studies, kinematic analysis, implementation framework, markerless, IMU, athlete monitoring

## Abstract

**Background:** Motion capture (MoCap) technologies have transformed athlete monitoring, yet athletic departments face complex decisions when selecting systems for multiple sports. **Methods:** We conducted a narrative review of peer-reviewed studies (2015–2025) examining optical marker-based, inertial measurement unit (IMU) systems, including Global Navigation Satellite System (GNSS)-integrated systems, and markerless computer vision systems. Studies were evaluated for validated accuracy metrics across indoor court, aquatic, and outdoor field environments. **Results:** Optical systems maintain sub-millimeter accuracy in controlled environments but face field limitations. IMU systems demonstrate an angular accuracy of 2–8° depending on movement complexity. Markerless systems show variable accuracy (sagittal: 3–15°, transverse: 3–57°). Environmental factors substantially impact system performance, with aquatic settings introducing an additional orientation error of 2° versus terrestrial applications. Outdoor environments challenge GNSS-based tracking (±0.3–3 m positional accuracy). Critical gaps include limited gender-specific validation and insufficient long-term reliability data. **Conclusions:** This review proposes a tiered implementation framework combining foundation-level team monitoring with specialized assessment tools. This evidence-based approach guides the selection of technology aligned with organizational priorities, sport-specific requirements, and resource constraints.

## 1. Introduction

The pursuit of peak athletic performance and injury resilience increasingly relies on sophisticated insights provided by sports scientists. Sport biomechanics stands as one of the major pillars of modern sport science, providing an objective foundation for analyzing the intricate subtleties of human movement that often go unnoticed to the naked eye yet can drive considerable performance gains—potentially elevating an athlete with an average ranking in competitions to podium status, or markedly reducing their injury risk through improved movement efficiency [1,2,3]. By applying principles from mechanics, anatomy, and physiology, biomechanists quantitatively analyze human movement to optimize technique, identify inefficiencies, and understand injury mechanisms [2,4]. Central to biomechanics analysis is motion capture (MoCap) technology, which translates the complex, dynamic movements of athletes into digital data, enabling detailed kinematic and, in some cases, kinetic analyses [5]. This objective data enables evidence-based technique refinement and injury prevention strategies, with MoCap applications in sports biomechanics expanding significantly over the past decade as technologies become more accessible, portable, and capable of capturing increasingly complex movements [6].

However, the rapid proliferation of commercial MoCap systems presents both opportunities and challenges for multi-sport organizations such as university athletic departments and national governing bodies. These organizations must navigate a complex landscape of competing vendor claims, varying levels of scientific validation, and considerable differences in cost, usability, and infrastructure requirements when selecting appropriate systems [7]. They must cater to the diverse biomechanical demands of various sports practiced in vastly different environments from the controlled setting of an indoor gymnasium to the unpredictable conditions of an outdoor field or aquatic facility [8].

The choice between established optical marker-based systems, portable Inertial Measurement Unit (IMU) systems, and emerging markerless computer vision-based systems involves complex trade-offs between accuracy, practicality, and cost-effectiveness. Optical systems offer exceptional accuracy but require controlled environments and an extensive setup [9]. IMU systems provide portability but may sacrifice measurement precision [10]. Markerless systems promise convenience and ecological validity but continue to evolve in accuracy and reliability [11]. In the meantime, multi-sport organizations have increasingly started to deploy these technologies for their athletes’ performance improvement and injury prevention [12,13]; however, they face distinct implementation challenges including resource allocation across diverse sporting environments and equipment coordination among multiple coaching staff members with varying levels of technical expertise. While comprehensive systematic reviews have examined MoCap technologies in sports contexts [5,8,14], none have addressed the unique decision-making challenges faced by multi-sport organizations requiring contextual interpretations of validation data, including accuracy metrics, ecological validity, and cost-effectiveness considerations within organizational frameworks.

A narrative review methodology was selected enabling the integration of both peer-reviewed validation evidence and real-world implementation considerations essential for organizational decision making. We synthesize evidence published between 2015 and 2025, focusing on validation studies in sports-relevant contexts, to address three fundamental questions: (1) Which MoCap technologies provide sufficient accuracy and reliability for specific sporting applications? (2) How do environmental factors affect system performance? (3) What implementation considerations should inform purchasing decisions?

Our approach systematically weighs technological capabilities against implementation constraints through a proposed cost–benefit framework, progressing from technology landscape comparisons of sport-specific applications to implementation framework guidance that translates technical capabilities into strategic recommendations aligned with organizational priorities and practical constraints.

## 2. Methods

We conducted comprehensive searches of PubMed, SPORTDiscus, and Web of Science databases from January 2015 to January 2025 using combinations of keywords including “motion capture,” “sports biomechanics,” “validation,” “accuracy,” “IMU,” “markerless,” “optical tracking,” and sport-specific terms. The initial search yielded 986 articles, with 953 remaining after duplicate removal (*n* = 33). The large initial pool reflects the broad scope necessary to capture validation studies across diverse sporting applications and technological approaches.

Studies were included if they met any one of the following criteria: (1) peer-reviewed studies validating motion capture systems in sports contexts or sports-related movements (jumping, running, throwing, or cutting); (2) comparative studies evaluating different motion capture technologies; (3) implementation reports from multi-sport organizations or athletic departments; OR (4) studies reporting quantitative accuracy metrics relevant to athletic performance assessment. This inclusive approach was necessary to capture the diverse validation evidence required for multi-sport implementation decisions, as few studies directly compare all three technology categories within identical sporting contexts. Exclusion criteria included the following: manufacturer-sponsored studies without independent validation, studies focusing exclusively on clinical populations without athletic relevance, conference abstracts without full peer-reviewed publications, and studies lacking quantitative performance metrics. For outdoor field sports validation, we included studies evaluating Global Navigation Satellite System (GNSS)-integrated tracking systems, as these represent primary solutions for large-area athlete monitoring in field environments. Studies were prioritized based on the following: (1) their relevance to multi-sport implementation scenarios, (2) methodological rigor of validation protocols, (3) recency of publication (studies from 2020 to 2025 were prioritized, given rapid technological advancements), and (4) availability of comparative accuracy data across different sporting environments.

From the initial 953 articles, titles and abstracts were screened for relevance to motion capture validation in athletic contexts, resulting in 234 articles for full-text review. Of these, 82 studies met eligibility criteria and provided sufficient methodological detail for synthesis.

## 3. Technology Landscape: Capabilities and Limitations

The field of MoCap for sports biomechanics is dominated by three primary technological approaches: optical marker-based systems, IMU systems, and markerless systems leveraging computer vision [5]. While hybrid approaches combining elements (e.g., IMU-supported markerless tracking) and multimodal systems integrating diverse sensor data with artificial intelligence (AI) are emerging [6,15], understanding the core characteristics, advantages, and limitations of these three main categories is fundamental for any organization considering MoCap adoption.

### 3.1. Optical Marker-Based Systems

Optical marker-based MoCap systems remain the gold standard for biomechanical analysis in sports science [5,16]. These systems function by using multiple synchronized cameras, typically emitting infrared light, to track the three-dimensional (3D) position of reflective markers placed on specific anatomical landmarks or segments of an athlete’s body. Through triangulation algorithms, these systems calculate the precise 3D coordinates of each marker over time, allowing for the reconstruction of segmental and joint kinematics [8].

Commercial systems such as Vicon (Oxford Metrics Group Ltd., Oxford, UK), Qualisys (Qualisys AB, Gothenburg, Sweden), and OptiTrack (NaturalPoint, Corvallis, OR, USA) offer sub-millimeter accuracy in controlled environments when properly calibrated. These systems are widely regarded as the ‘gold standard’ for their validated kinematic measurement accuracy in controlled laboratory environments, forming a benchmark against which newer technologies are often validated [9].

Recent innovations have improved the portability and ease of setup of optical systems. For example, the Raptor-E system (Motion Analysis Corporation, Rohnert Park, CA, USA) offers enhanced outdoor performance with cameras designed to filter ambient infrared radiation, enabling data collection in varied lighting conditions [5]. Similarly, OptiTrack’s Prime series cameras provide increased capture volumes suitable for analyzing movements like volleyball jumps or basketball shots within realistic playing environments.

#### 3.1.1. Accuracy and Precision Metrics

The primary advantage of optical systems lies in their exceptional accuracy and precision. Manufacturers and independent validation studies report sub-millimeter positional accuracy under optimal conditions. For instance, Vicon systems have demonstrated static mean absolute errors of 0.15 mm and dynamic errors below 2 mm, potentially as low as 0.3 mm depending on the marker size and sampling rate [17]. Similarly, OptiTrack systems typically achieve measurement errors below 0.2 mm, even in large volumes, with errors below 0.1 mm possible in smaller areas. One study using 42 OptiTrack cameras in a 135 m^3^ volume found 97% of the area had errors below 200 μm [8].

The high fidelity of optical systems, combined with their high sampling rates (often >200 Hz), makes them ideal for analyzing the fast, complex movements seen in sports like gymnastics and for research requiring precise joint angle calculations. Furthermore, these systems are readily integrated with other biomechanics tools like force plates and electromyography (EMG) systems, allowing for synchronized kinetic and neuromuscular data collection [16].

#### 3.1.2. Limitations and Practical Constraints

Despite their accuracy, optical systems suffer from significant practical field-based limitations. They typically require a dedicated laboratory space with a controlled background and lighting conditions to minimize reflections and ensure marker visibility [16,18,19]. Environmental reflections present particular challenges in indoor court sports, in which highly reflective gymnasium floors and polished surfaces create infrared reflections that interfere with marker detection algorithms, leading to false positives and tracking errors [20]. While threshold adjustments and camera masking can partially mitigate these issues, they often require facility modifications such as anti-reflective matting or strategic camera positioning.

Marker occlusion, in which one body part or object blocks the cameras’ view of a marker, is a common problem that can lead to data loss or inaccuracies, particularly in team sports or activities involving close interaction or equipment [16,18,19]. Additionally, setup and calibration procedures are time-consuming (approximately 30–60 min), requiring trained personnel for precise marker placement [21]. The need to attach markers to athletes’ skin can potentially alter their natural movement patterns, raising ecological validity concerns and making these systems impractical for routine multi-team monitoring [22].

The substantial cost of multi-camera systems, associated software, and a dedicated laboratory space often places them beyond smaller organizations’ reach. While large capture volumes are achievable, they require increased investment and complexity, limiting their practical implementation across diverse sporting environments.

### 3.2. Inertial Measurement Unit (IMU) Systems

IMU-based systems utilize small, lightweight sensors worn directly on the athlete’s body segments [4]. Each sensor typically contains a triad of accelerometers, gyroscopes, and magnetometers. Accelerometers measure linear acceleration, gyroscopes measure angular velocity, and magnetometers provide the orientation relative to the Earth’s magnetic field. Sophisticated sensor fusion algorithms integrate data from these components, often across multiple sensors in a network, to estimate segment orientation and, subsequently, joint kinematics [23,24]. Some of the leading commercial systems include Xsens (Movella, Enschede, Netherlands), Noraxon’s IMU-only sensors (Noraxon, Scottsdale, AZ, USA), and APDM Opals (Clario, Portland, OR, USA).

#### 3.2.1. Accuracy Compared to Optical Systems

IMU systems face inherent accuracy limitations compared to optical systems, particularly concerning absolute position and certain joint rotations [5]. Sensor drift (the accumulation of small errors over time) can affect their accuracy during long captures, although advanced algorithms aim to minimize this [25]. While their orientation accuracy can be high, deriving precise joint angles, especially in multiple planes, remains challenging.

Validation studies comparing IMUs (e.g., Xsens) to optical systems (e.g., Vicon) often report root mean square errors (RMSEs) for joint angles that vary considerably depending on the joint, movement plane, and task complexity. For example, while walking and running, Xsens showed an acceptable RMSE for knee flexion/extension (~3.2°) and ankle dorsi-/plantarflexion (~4.5°), but larger errors for hip flexion/extension (~10.1°) [24]. Another study comparing APDM Opals to Vicon during dynamic tasks (jumps, squats, lunges, and cuts) found sagittal plane RMSEs ranging from 8.11° to 28.37°, frontal plane RMSEs from 3.26° to 16.98°, and highly variable transverse plane RMSEs from 5.08° to 116.75° [17].

Accuracy can also be compromised by improper sensor placement, inadequate calibration procedures, and ferromagnetic interference in the environment [10]. Furthermore, specific biomechanical measures, such as the base of support (BOS) derived from foot position, can exhibit considerable errors, particularly during movements involving knee flexion [26].

#### 3.2.2. Advantages and Practical Applications

The key advantage of IMUs is their portability and flexibility [27]. Unconstrained by external cameras or specific lighting conditions, they allow MoCap to be performed in ecologically valid settings—on the field, court, track, or even in the water (with appropriate waterproofing) [28]. Their setup is generally faster than that of optical systems, primarily involving strapping sensors to the athlete. They can capture data over large areas and extended durations, making them well suited for monitoring training load, counting repetitions (e.g., laps, strokes, and jumps), assessing temporal parameters, and analyzing activities for which lab-based capture is impractical, such as skiing or rowing [7]. Some modern systems also incorporate algorithms to mitigate magnetic disturbance, enhancing their usability in varied environments.

Single-unit IMU systems provide valuable workload monitoring capabilities for team-based applications. These systems typically include 20–30 units that can be shared across teams with different training schedules, offering a cost-effective solution for multi-sport departments [29]. Single-unit systems excel at load management metrics including activity counts, basic temporal parameters, and movement intensity estimates rather than detailed joint kinematics.

Multi-sensor IMU systems provide richer kinematic data for detailed biomechanical analysis but require greater expertise and a longer setup time. Xsens MVN has been validated for gymnastics, swimming, track and field, and volleyball with a joint angle accuracy of 2–8° depending on movement complexity [30,31,32]. These systems derive joint angles from segment orientation measurements, with accuracy varying by anatomical plane (typically, it is the highest in sagittal planes and the lowest in transverse planes). Multi-sensor configurations enable comprehensive full-body kinematic analysis suitable for technique assessment and injury risk screening applications.

### 3.3. Markerless Systems

Markerless MoCap utilizes computer vision techniques, increasingly powered by deep learning and artificial intelligence, to track human movement directly from video footage without physical markers or sensors [5,14,33,34]. These systems employ multiple synchronized cameras (though it is possible to use a single camera) to detect body landmarks in 2D images and reconstruct 3D positions through biomechanical model fitting [11,22]. Commercial examples targeting sports and biomechanics include Theia 3D (Kingston, ON, Canada), Kinetrax (Boca Raton, FL, USA), DARI Motion (Overland Park, KS, USA), and Simi Motion’s (Unterschleissheim, Germany) markerless solutions, while open-source tools like OpenCap (Stanford, CA, USA) and platforms like DeepLabCut and OpenSim (Stanford, CA, USA) are also gaining traction [19].

Multi-camera systems achieve RMSEs of 3–15° in the sagittal plane, while single-camera systems may exhibit errors exceeding 20° for complex movements [11,22,35]. Single-camera systems, predominantly utilizing depth sensors like Microsoft Kinect, demonstrate a substantially inferior performance compared to that of multi-camera configurations, particularly in terms of the joint angle error in frontal and transverse planes [21].

OpenCap represents a significant advancement in accessibility, reducing equipment costs by approximately 215-fold compared to traditional laboratory systems [35]. However, smartphone camera specifications (typically with 60Hz sampling rates) present fundamental limitations for high-velocity athletic applications requiring >200 Hz sampling rates for explosive movements [36]. Additionally, requirements for controlled lighting and uncluttered backgrounds limit its applicability in dynamic team sport environments [35].

The true ‘cost’ of open-source systems often manifests in staff time and delayed feedback to coaches, potentially offsetting initial financial savings. However, for research-focused departments with strong technical expertise, these systems can provide valuable customization opportunities not available in commercial packages.

#### 3.3.1. Accuracy and Reliability Assessments

Despite rapid advancements, the accuracy and reliability of markerless systems remain key concerns and areas of active research and validation [22]. Current systems often exhibit a lower accuracy than optical systems, particularly for precise joint center localization, transverse plane rotations (e.g., hip internal/external rotation), and movements involving a high speed, great complexity, or significant occlusion [37].

A systematic review found that while spatiotemporal parameters (like speed and step length) showed good-to-excellent agreement with marker-based systems during gait tests, kinematic agreement was moderate-to-excellent for the hips and knees (especially in the sagittal plane) but poor for the ankles [11]. A validation of Theia3D against Vicon showed sagittal plane RMSEs of 3.20–15.66° and frontal plane RMSEs of 2.12–9.14°, but the transverse plane RMSEs ranged widely from 3.16° to 56.61° [22].

System performance is highly dependent on numerous factors: the quality and sophistication of the underlying pose estimation algorithms (often proprietary “black boxes”), the quality and diversity of the data used to train these algorithms, the number, positioning, and specifications of the cameras used, lighting conditions, the athlete’s clothing (loose clothing may be problematic in some systems’ analyses), and the complexity of the background environment [38].

#### 3.3.2. Advantages and Implementation Considerations

The primary appeal of markerless systems lies in their potential to combine the non-invasive nature of IMUs with the potential for a higher spatial accuracy derived from cameras, while eliminating the need for markers altogether [5]. This significantly reduces participant preparation time and burden, potentially allowing for more natural movement patterns. However, the system setup time for camera positioning, calibration, and environmental optimization can still be substantial when deploying it to new locations, often requiring 20–45 min depending on the number of cameras and environmental complexity [11,22]. This setup consideration becomes particularly relevant for organizations requiring frequent deployment across multiple venues.

Markerless technology opens possibilities for analyzing large cohorts of athletes efficiently, assessing movement in realistic contexts (including potential in-game scenarios), and even analyzing historical video footage [39]. Markerless systems offer particular advantages in environments with highly reflective surfaces, such as polished gymnasium floors, where optical marker-based systems typically struggle with infrared reflections that can interfere with marker detection. Unlike optical systems that rely on infrared illumination, markerless systems using visible light cameras are less susceptible to these reflection artifacts, though they may still face challenges with specular highlights and lighting variations.

The clear progression towards more portable and user-friendly systems (IMU and markerless systems) reflects a strong demand within sports science and practice for tools that enable data collection in ecologically valid environments, moving beyond the constraints of traditional laboratories [7]. This shift acknowledges that athlete movement and performance can differ significantly between sterile lab settings and the dynamic, often unpredictable, conditions of actual training sessions and competitions.

A limitation of some markerless systems is their inability to simultaneously track sports implements and equipment alongside human movement. Most markerless systems, including Theia3D, focus exclusively on human pose estimation and cannot track objects such as bats, rackets, balls, or other sports equipment. This represents a critical gap for sports requiring implement analysis, such as baseball batting mechanics, tennis serve analysis, or golf swing assessment, in which understanding the relationship between athlete kinematics and equipment dynamics is essential for performance optimization. While some specialized systems like Kinetrax have demonstrated they are able to track baseballs in controlled environments, comprehensive athlete–implement interaction analysis remains largely unavailable in current markerless solutions.

### 3.4. Soft Tissue Artifacts Across Motion Capture Technologies

All MoCap systems discussed measure skin-mounted markers or sensors rather than actual bone kinematics, introducing systematic soft tissue artifacts. Validation studies using bone–pin references demonstrate substantial errors: optical marker-based systems exhibit an RMSE of 6.8–11.2° for hip flexion and 3.2–8.9° for knee flexion while walking, with errors increasing during dynamic movements [40,41]. IMU systems face compounded effects from translational and rotational soft tissue motion, often showing higher RMSE values during dynamic tasks than maker-based systems. These artifacts significantly impact multi-sport organizations’ data interpretation. Organizations should implement standardized procedures, prioritize movement-specific validation, and apply conservative interpretation thresholds that account for known artifact magnitudes to avoid the over-interpretation of biomechanical differences falling within expected measurement error ranges.

### 3.5. Comparative Analysis of Motion Capture Technologies

Table 1 provides a comparative overview of the three primary MoCap technologies, highlighting their key characteristics, advantages, limitations, and typical applications in sports biomechanics.

## 4. Sport-Specific Applications

The application of MoCap technology varies significantly across different sporting environments, each presenting unique challenges and validation considerations. This section examines how various MoCap systems perform in three primary sporting contexts: indoor court sports, aquatic environments, and outdoor field settings. For each environment, we analyze system performance based on independent validation studies, identify key implementation challenges, and provide evidence-based recommendations for optimal technology selection.

### 4.1. Indoor Court Sports

Indoor court sports (e.g., basketball, volleyball, and handball) present unique challenges for MoCap systems due to their combination of high-velocity movements, rapid direction changes, and frequent player interactions within relatively confined spaces [5]. These sports require systems capable of capturing explosive multi-directional movements while maintaining accuracy during player interactions and complex maneuvers.

Validation studies comparing different technologies in indoor court applications have yielded important insights regarding their relative performance. Markerless AI-driven systems have shown promising results in controlled validation studies. Aleksic et al. (2024) reported that MMPose achieved root mean square error (RMSE) values as low as 0.021–0.022 m for center of mass tracking and intraclass correlation coefficient (ICC) values above 0.91 for temporal jump variables when compared to Qualisys optical systems [42]. While markerless systems slightly overestimated jump height, they maintained practical reliability thresholds (ICC > 0.97) for most applications.

For tracking-focused applications, Kinexon’s Local Positioning System (LPS), validated against Qualisys, demonstrated a position RMSE of 8–9 cm and a speed RMSE of 0.07 m/s, with higher errors observed during high-velocity movements [29]. This level of accuracy is generally sufficient for tactical and workload monitoring but may be inadequate for detailed technical analysis of specific movements.

IMU-based systems have shown an angular accuracy within ±2–8° for most joint angles in indoor court movements, with performance remaining relatively consistent across both genders, though most studies reported limited female representation in their validation cohorts [42]. This gender imbalance represents a significant gap in the validation literature that warrants attention in future research.

#### 4.1.1. Implementation Challenges

Indoor court sports present several significant challenges for MoCap implementation:

1. Capture Volume Constraints: Standard court dimensions (e.g., 28 × 15 m for basketball) frequently exceed optimal capture volumes for most optical systems, necessitating either multiple camera setups or compromises in coverage area [18];
2. Movement Complexity: The rapid, multi-directional movements characteristic of these sports, such as cutting, jumping, and landing, generate great accelerations that challenge sensor sampling rates and filtering algorithms [29];
3. Player Interactions: Physical contact between athletes affects both marker visibility in optical systems and sensor stability in IMU-based approaches, potentially introducing data artifacts or gaps [18];
4. Sport-Specific Movements: Basketball shooting mechanics, volleyball blocking jumps, and handball throwing motions each present unique validation requirements that must be addressed with specialized protocols [5].

#### 4.1.2. Recommended Approaches

**Primary System**: Multi-camera markerless systems (e.g., Theia3D and DARI Motion)

- Minimal athlete preparation; no markers required, reducing workflow disruption;
- Validated RMSE: 0.021–0.022 m for center of mass tracking; ICC > 0.91 for temporal jump variables vs. Qualisys optical system [42];
- Setup time under 30 min per venue; enables rapid deployment for multiple teams;
- Maintains an ICC > 0.97 for most temporal variables, even with a slight jump height overestimation;
- Best for team-wide screening and routine technical analyses for which efficiency is critical.

**Secondary System**: IMU-based systems (e.g., Xsens MVN and Noraxon)

- Enhanced joint angle accuracy (±2–8°) for detailed biomechanical assessment [32,33];
- Consistent angular accuracy across genders; limited female validation data [32];
- Portable; suitable for field-based or on-court assessments without lab infrastructure;
- Requires individual sensor attachment and calibration, which may disrupt sessions;
- Optimal for specific technique analysis and injury risk screening, not team-wide monitoring.

**Specialized Applications**: Multi-camera optical systems

- Maximum precision (static mean absolute error: 0.15 mm; dynamic error <2 mm) in controlled environments [21,43,44];
- Requires dedicated lab space, controlled lighting, and infrastructure modifications;
- High initial investment; justified for research or clinical applications needing gold-standard accuracy;
- Can be integrated with force plates and EMG for comprehensive biomechanical analysis.

This tiered combination provides both the precision needed for detailed biomechanical analysis and the practicality required for routine monitoring in dynamic team sport environments.

### 4.2. Aquatic Sports

Aquatic environments, encompassing swimming and diving, pose unique and significant challenges for all types of MoCap technology [45]. The presence of water introduces issues of sensor waterproofing, light refraction (which affects cameras), marker adhesion and visibility, signal attenuation for wireless transmission, and the practical difficulty of positioning equipment around a pool.

Optical systems, while used for detailed stroke analysis in specialized flumes or limited pool sections, face substantial hurdles in calibration due to light bending at the air–water interface and maintaining marker visibility [5]. Consequently, IMUs have gained considerable traction in aquatic sports research and monitoring.

Studies evaluating waterproof inertial systems have provided important insights into their performance in aquatic environments. The Cometa WaveTrack and OPAL APDM inertial systems demonstrated a mean dynamic orientation error of 6.1° in water compared to 4.4° in dry laboratory conditions, highlighting a consistent 2° degradation attributable to aquatic interference [46]. This degradation must be considered when interpreting kinematic data collected in water.

Systematic biases have been observed in swimming velocity estimation, with IMU-derived values consistently underestimating gold-standard speedometer measurements (*p* < 0.001) [45]. This underscores the importance of sport-specific validation rather than relying on terrestrial performance metrics when evaluating systems for aquatic use.

For event detection and classification, IMU systems have shown impressive results. Studies report high accuracy for the automatic detection of swimming parameters such as lap counting (99.7–100%) and style classification (>98% sensitivity/specificity or F1 scores > 0.96) using machine learning or deep learning models [46]. However, obtaining accurate, detailed 3D joint kinematics comparable to terrestrial applications remains challenging due to the complex dynamics of water resistance, sensor placement limitations, and potential signal issues.

Diving presents distinct biomechanical challenges that differ substantially from swimming applications. The sport’s characteristic rapid rotations reaching 1440°/s during platform dives [47] combined with brief flight durations of 1.5–2.5 s require high sampling rates (a minimum of 100–250 Hz) to capture sufficient data points for accurate kinematic analysis [48]. Camera positioning introduces unique complications, as capturing the complete sequence from the approach to entering the water requires coverage exceeding 10 vertical meters. This often necessitates upward-facing camera angles that result in direct exposure to facility lighting, causing sensor saturation and compromising image quality. The extreme viewing angles reduce marker visibility to below 50% in optical systems and significantly challenge pose estimation algorithms in markerless approaches, particularly for tucked positions in which self-occlusion is prevalent [49].

Current diving biomechanics research predominantly relies on multiple synchronized high-speed cameras with manual digitization, as validated automatic tracking solutions remain limited. While IMU systems show promise for measuring rotational kinematics during flight phases, their accuracy degrades during water entry due to extreme accelerations exceeding typical sensor ranges (±16 g) and synchronization challenges for identifying critical events such as the last point of contact and water entry [50]. These technical limitations highlight the need for specialized MoCap solutions specifically designed for diving’s unique spatiotemporal and environmental demands.

#### 4.2.1. Implementation Challenges

Aquatic sports present several significant validation challenges for MoCap systems:

1. Water Resistance Effects: Water fundamentally alters both athlete movement patterns and sensor performance, creating unique dynamics not present in terrestrial environments [45];
2. Magnetic Interference: Pool infrastructure (particularly stainless steel components) creates magnetic disturbances that affect IMU orientation estimates [46]. The OPAL APDM system maintained a robust performance, provided that the sensors were positioned at least 100 cm from the pool walls to mitigate this interference;
3. Signal Transmission: Water significantly attenuates wireless signals, making real-time data collection difficult and often necessitating on-board storage with post-session downloading [46];
4. Waterproofing Requirements: Sensors must maintain performance integrity while submerged without compromising athlete comfort or increasing drag, presenting a complex design challenge [45].

#### 4.2.2. Recommended Approaches

**Primary System:** Waterproof IMU systems with specialized mounts

- The only practical solution for continuous in-water monitoring with an acceptable level of degradation in accuracy;
- Mean dynamic orientation error of 6.1° in water vs. 4.4° when dry; consistent aquatic degradation of ~2° [46];
- High event detection accuracy: 99.7–100% lap counting; >98% style classification sensitivity/specificity [46];
- Maintains a robust performance if the sensors are ≥100 cm from the pool walls to minimize magnetic interference;
- Enables stroke analysis and monitoring without disrupting natural swimming patterns.

**Secondary System:** Underwater camera systems

- Provides visual technique analysis in controlled settings for detailed stroke mechanics;
- Millimeter accuracy achievable with proper calibration [51]; typically 2D unless using specialized multi-camera setups;
- Requires significant infrastructure and expertise in underwater calibration and air–water interface correction;
- Best for controlled stroke analysis, not routine monitoring.

**Magnetic Interference Mitigation:** Implementation strategy

- Pool infrastructure (stainless steel) can cause significant IMU orientation errors;
- OPAL APDM: robust if sensors are ≥100 cm away from metal infrastructure;
- Failure to mitigate can increase the heading RMSE from <5° to 17° [52];
- Sensor placement protocols must address pool components and electronics.

For stroke analysis and lap timing, single-sensor IMU systems achieve practical feasibility with a slightly reduced accuracy compared to multi-sensor configurations. These systems, typically worn on the wrist, sacrum, or back, provide automated swimming style detection, lap and stroke counting, lap time measurement, and basic parameter estimation including speed and stroke rate/length.

### 4.3. Outdoor Field Sports

Outdoor field sports (e.g., soccer, rugby, and American football) present the greatest challenges for MoCap validation due to large playing areas, variable environmental conditions, and complex player interactions [5]. Variable and often bright sunlight, shadows, uneven surfaces, large open spaces, unpredictable weather conditions, and potential sources of magnetic interference create significant hurdles for accurate motion tracking.

Traditional optical marker-based systems are generally impractical for outdoor field sports due to the extensive setup required, sensitivity to ambient light, and the difficulty of covering large areas with a sufficient camera density [8]. This leaves IMUs and markerless systems as the primary options for capturing kinematics data in these settings.

IMUs are widely adopted in outdoor sports, leveraging their portability and independence from external infrastructure [7]. They are commonly integrated into GPS units or used as standalone sensors to monitor athlete load, distance covered, speed profiles, impacts, and basic gait characteristics during training and competitions. Validation studies have shown that Global Navigation Satellite System (GNSS)-integrated IMU systems with dual-frequency or RTK capabilities can achieve a positional accuracy of ±0.3 m under optimal conditions, though this degrades in challenging environments such as stadiums with high stands that can block satellite signals [29].

Markerless systems hold great promise for outdoor sports due to their non-invasive nature, but they face substantial hurdles in field settings [22]. Fluctuating sunlight and shadows can drastically affect image quality and algorithm performance. Complex, dynamic backgrounds and the presence of multiple players increase the difficulty of accurate athlete detection and tracking, leading to potential identity swaps or data loss. Maintaining tracking accuracy over the large distances involved in field sports is also challenging.

Despite these challenges, specialized markerless systems have been developed and validated for specific outdoor applications. Kinetrax, for example, has been used extensively in professional baseball for in-game analysis of pitching and hitting biomechanics, employing high-speed cameras and custom neural networks trained for specific ballpark environments to optimize accuracy [19]. Similarly, the pitchAI system showed reasonable validity for analyzing pitching kinematics from a single camera in an outdoor setting, though with limitations in tracking the glove arm due to occlusion issues. Importantly, these successful outdoor implementations operate in controlled, predictable locations (pitching mounds and batting boxes) rather than across dynamic field environments, highlighting the current limitations of markerless systems for truly unrestricted outdoor sports applications.

#### 4.3.1. Implementation Challenges

Outdoor field sports present several unique challenges for MoCap implementation:

1. **Environmental Variability**: Changing light conditions, shadows, and weather (rain and wind) can significantly impact system performance, particularly for optical and markerless approaches [22];
2. **Extended Capture Volumes**: The large playing areas in field sports (e.g., soccer fields up to 120 × 90 m) exceed the practical coverage of most camera-based systems without significant compromises in resolution or accuracy [18];
3. **GNSS Signal Limitations**: Stadium architecture, tree coverage, and other obstructions can degrade GNSS signal quality, affecting the accuracy of position tracking in integrated IMU-GNSS systems [29];
4. **Multi-Player Tracking**: Distinguishing between multiple athletes in team settings, particularly during close interactions or when wearing similar uniforms, presents significant challenges for automated tracking systems [18].

#### 4.3.2. Recommended Approaches

**Primary System:** GNSS-integrated IMU systems with dual-frequency or RTK (e.g., Catapult Vector and STATSports Apex) capabilities

- The only solution for team-wide monitoring over large areas (up to 120 × 90 m);
- Positional accuracy: ±0.3 m under optimal conditions; ICC > 0.80 for key metrics [29,53];
- Validated for total distance, speed, positional coordinates, and acceleration counts in field studies;
- Performance confirmed for basic kinematics during training and competitions;
- Accuracy degrades in challenging environments (in stadiums and in locations with trees); site-specific validation is required.

**Secondary System:** Portable markerless multi-camera systems (e.g., Theia3D, OpenCap)

- Enables detailed technique analysis in specific field zones (kicking and throwing);
- Theia3D: a sagittal RMSE of 3.20–15.66° and a frontal RMSE of 2.12–9.14° [22];
- Commercial systems offer established workflows; open-source (OpenCap) requires technical expertise and independent validation;
- Best for specific movement analysis and not continuous field monitoring due to setup complexity.

**Specialized Applications:** Fixed installation markerless systems (e.g., Kinetrax)

- Permanent installations allow repeated analyses in controlled locations (e.g., pitching mounds);
- Validated for professional baseball using high-speed cameras and custom neural networks [18];
- Optimized accuracy via environment-specific training/calibration;
- Limited to controlled locations, not dynamic field environments.

Organizations should verify vendor claims by carrying out an independent validation, as significant discrepancies exist between manufacturer specifications and field performance, particularly for positional accuracy in challenging environments [54].

### 4.4. Comparative Analysis of Environmental Factors

Table 2 provides a comparative analysis of MoCap system performance across different sporting environments, synthesizing findings from independent validation studies. This information can guide technology selection based on the specific environmental conditions and performance requirements of different sports.

The validation of any MoCap system must be considered in the context of the specific task it is intended to measure. Findings from studies analyzing relatively slow, controlled movements like walking gait cannot be directly extrapolated to predict performance during highly dynamic, unpredictable sporting actions like cutting or tackling. Accuracy often degrades as movement speed and complexity increase. Therefore, organizations must prioritize validation data that closely matches their specific sport(s) and the key movements they aim to analyze. Relying solely on manufacturer specifications or validation from unrelated tasks carries a significant risk of adopting a system that fails to meet practical needs.

## 5. Implementation and Recommendation Framework for Athletic Organizations

The successful implementation of MoCap technology in multi-sport settings depends primarily on aligning systems with specific organizational priorities, resources, and sport-specific requirements. This section provides a structured framework for selecting appropriate MoCap technologies based on organizational focus, sport-specific needs, and cost–benefit considerations, incorporating both evidence-based validation findings and practical implementation strategies.

### 5.1. Strategic Approaches Based on Organizational Focus

Athletic departments typically approach MoCap with one of several primary objectives, each warranting different technological solutions tailored to their specific goals and constraints. It is crucial to emphasize that both performance optimization and injury prevention applications require equally high kinematic precision for meaningful biomechanical analysis. The fundamental accuracy requirements for measuring joint angles, velocities, and accelerations are dictated by the biomechanical phenomena being studied, not the application domain [29,55]. The recommendations that follow address practical implementation considerations, specifically the trade-offs between precision, portability, and workflow integration, rather than suggesting different accuracy standards.

#### 5.1.1. Performance Optimization Focus

Organizations primarily interested in enhancing technical performance across multiple teams should consider portable IMU-based systems as their foundation. These systems offer adequate precision for field-based performance monitoring while providing the workflow efficiency required for routine team monitoring and the flexibility to monitor numerous athletes across diverse environments [5]. For detailed technical analysis requiring the maximum precision, this foundation should be supplemented with laboratory-grade systems, similar to injury prevention protocols. For example, a collegiate athletic department monitoring pitching mechanics across baseball and softball could effectively deploy single-unit IMU systems like Catapult Vector, which has demonstrated ICC values >0.80 for key performance metrics including speed, distance covered, acceleration patterns, and movement counts in field validation studies [53]. For pitching-specific applications, single IMUs would primarily focus on workload metrics such as throw count, arm speed estimates, activity duration, and basic temporal parameters rather than detailed biomechanical variables like joint angles or kinetic measures [56,57].

While not matching optical systems’ joint angle precision, IMUs provide reliable workload monitoring and technique consistency assessment with minimal setup disruption to training sessions. For instance, Lapinski et al. (2019) validated a wearable IMU system for capturing baseball pitching biomechanics, finding it capable of measuring key performance parameters with sufficient accuracy for practical applications while allowing athletes to perform in their natural environment [10]. This tiered implementation strategy enables organizations to balance routine monitoring capabilities with periodic in-depth analysis, optimizing both practical implementation constraints and biomechanical insight quality [5,58]. This strategic approach aligns with established practices in sports science in which periodic comprehensive assessments complement routine monitoring protocols, as demonstrated in multi-modal motion capture implementations that combine different technologies to maximize accuracy while maintaining practical feasibility [5]. For more detailed technical analysis of specific high-value athletes or complex movements, this foundation can be supplemented with periodic laboratory assessments using optical systems or advanced markerless solutions [59].

#### 5.1.2. Injury Prevention and Return-to-Play Focus

Organizations emphasizing injury risk screening and rehabilitation monitoring require controlled measurement environments that enable maximum precision utilization, particularly for specific injury-relevant movements. For such settings, multi-sensor IMU systems (e.g., Xsens MVN) are recommended for field-based assessments, as they have demonstrated an angular accuracy of 2–8° depending on movement complexity [32]. These systems can effectively track running gait parameters, jump mechanics when landing, and sport-specific movements outside laboratory settings.

The addition of a mid-range optical system (six to eight cameras) for detailed baseline assessments and return-to-play validation testing can further enhance clinical decision making. Thewlis et al. (2013) demonstrated that even mid-range optical systems with six to eight cameras can provide a comparable spatial accuracy to high-end systems for most clinical applications, making them a cost-effective option for athletic departments [60].

Several professional sports organizations have successfully implemented this hybrid approach, using portable IMU systems for daily monitoring and a dedicated motion laboratory with optical tracking for comprehensive assessments during the pre-season and rehabilitation phases. This strategy provides the detailed kinematic assessment necessary for clinical decision making while maintaining a reasonable level of cost efficiency [9].

#### 5.1.3. Multi-Sport Flexibility Requirements

Athletic departments overseeing diverse sports programs face unique challenges in technology selection due to the varied environments and biomechanical demands across different sports. A comprehensive approach addressing these diverse needs requires the strategic deployment of complementary technologies.

For a specified training room or sport movement testing facility, markerless MoCap systems such as Theia3D offer significant advantages. These systems can be prepared before each team’s training sessions or tests to collect accurate kinematic and performance data (e.g., jump height) with minimal setup time. Aleksic et al. (2024) validated such markerless systems for vertical jump analysis, finding strong agreement with force plate measurements (*r* = 0.992) and a high reliability (ICC > 0.9) for most temporal variables [42].

Most MoCap technologies can integrate with Athlete Management Systems (AMS) such as Teamworks, though markerless systems like Theia3D offer streamlined integration by outputting analysis-ready skeletal data, while optical systems typically require additional post-processing through software like Visual3D (C-Motion, Inc., 2025) before AMS integration [61]. The primary advantage of markerless systems for AMS integration lies in their direct output of processed kinematic data, reducing the technical expertise and time required for data pipeline management compared to marker-based systems that often require extensive post-collection processing workflows. For team monitoring during practices, IMU systems like Xsens MVN or Kinexon can serve as secondary systems, enabling the tracking of workloads, jump counts, and basic movement patterns across multiple athletes simultaneously [4].

#### 5.1.4. Precision vs. Practicality in Implementation

The apparent differences in technology recommendations between performance and injury applications reflect practical implementation considerations rather than precision requirements [16,29]. Performance coaches typically require systems that can be rapidly deployed across diverse training environments while providing immediate, actionable feedback during practice sessions. In contrast, sports medicine personnel often operate within dedicated facilities where controlled measurement conditions enable the optimal utilization of high-precision systems. Both applications benefit equally from the maximum available precision when biomechanical analysis is the primary objective.

### 5.2. Environment-Specific Implementation Strategies

Different sports environments present unique challenges for MoCap implementation, requiring tailored approaches to ensure optimal data quality and practical usability.

#### 5.2.1. Indoor Court Implementation

For indoor court sports (basketball, volleyball, and handball), key considerations include camera positioning to minimize blind spots and developing rapid setup protocols to integrate with practice schedules. Kinexon’s Local Positioning System (LPS) has demonstrated a position RMSE of 8–9 cm and a speed RMSE of 0.07 m/s in an independent validation test, making it suitable for tracking player movements in team settings [29].

Multi-camera markerless systems (e.g., Theia3D and DARI Motion) are recommended for team-wide screening and technique analysis when the setup time is critical. These systems provide sufficient accuracy for most technical analyses while minimizing athlete preparation time. For detailed biomechanical assessments of specific techniques or movements requiring higher precision, IMU-based systems (e.g., Xsens MVN and Noraxon) offer an enhanced joint angle accuracy when properly calibrated [24].

Magnetic interference can affect IMU accuracy in indoor environments, particularly from metal infrastructure (e.g., steel beams and lighting systems) and electronic equipment (e.g., scoreboards and sound systems). Studies have shown that the RMSE in the heading direction can increase from <5° to 17° under magnetic disturbance [52]. To mitigate this, IMU systems should be positioned away from metal infrastructures when possible, and sensor fusion algorithms with advanced filtering can help reduce interference effects. Modern IMU systems increasingly incorporate algorithms to mitigate magnetic disturbance, enhancing their usability in varied indoor environments [62].

#### 5.2.2. Outdoor Field Implementation

For outdoor and field sports such as soccer, rugby, American football, and lacrosse, GNSS-integrated IMU systems with dual-frequency or RTK capabilities are recommended for team-wide monitoring. Catapult Vector and STATSports Apex offer validated solutions with a positional accuracy of ±0.3 m (with RTK) and consistent ICC values >0.80 for fundamental tracking metrics including velocity, distance, positional coordinates, and basic kinematic parameters [7].

Portable markerless multi-camera systems (e.g., Theia3D and OpenCap) can serve as secondary systems for technique analysis in key areas (e.g., kicking zones and throwing areas). For permanent facilities in which specific movements are repeatedly performed in the same location (e.g., baseball pitching mounds and batting cages), fixed installation markerless systems like Kinetrax have demonstrated success in baseball applications with good agreement for throwing mechanics compared to optical systems [63].

Field sports present significant challenges related to environmental variability, extended capture volumes, and GNSS signal limitations. Organizations should verify vendor claims with independent validation studies, as significant discrepancies exist between manufacturer specifications and field performance, particularly for positional accuracy in challenging environments [55].

#### 5.2.3. Aquatic Implementation

For swimming, water polo, and other aquatic sports, waterproof IMU systems with specialized mounts are the recommended primary technologies. OPAL APDM and Cometa WaveTrack provide validated solutions for stroke analysis and performance monitoring in aquatic environments [45]. Underwater 3D optical systems are available from manufacturers like Qualisys, offering specialized underwater cameras (Miqus Underwater and Arqus Underwater) capable of full 3D motion capture in aquatic environments. These systems can achieve sub-millimeter accuracy underwater and be integrated with above-water systems for comprehensive analyses across air–water interfaces. However, implementation requires significant infrastructure investment and expertise in underwater calibration procedures [51].

Aquatic implementations must address unique challenges, including water resistance effects on movement patterns, magnetic interference from pool infrastructure, accounting for water entry or splashing in diving, and signal transmission limitations through water. Validation studies have demonstrated a mean dynamic orientation error of 6.1° in water versus 4.4° in dry conditions using the same systems, highlighting the importance of aquatic-specific validation [46]. To optimize data quality, sensors should be positioned at least 100 cm from pool walls and metal infrastructure to minimize magnetic interference.

### 5.3. Tiered Acquisition Strategy

Rather than purchasing all technologies simultaneously, staged implementation allows for capacity building, evaluations, and strategic expansion based on their demonstrated value and one’s identified needs.

#### 5.3.1. Foundation Phase (Years 1–2)

The initial phase should focus on a portable system that meets the immediate requirements of the highest-priority sports. For most organizations, this means investing in either of the following:

1. A team-based IMU system (e.g., Catapult or STATSports) for workload monitoring and basic movement analysis across multiple teams; these systems excel at load management and injury prevention rather than detailed biomechanical assessment;
2. A multi-camera markerless system for detailed technique analysis in a controlled environment, which provides precise joint kinematics for technical analysis.

These complementary approaches allow organizations to establish foundation-level monitoring before expanding to specialized applications. As such, this approach allows organizations to develop expertise, establish workflows, and demonstrate value before committing to more extensive investments [55].

Technology evolution timelines should inform these investment decisions, as markerless systems powered by AI and deep learning are rapidly approaching optical system accuracy for many applications. While optical systems currently maintain precision advantages for detailed kinematic analysis, organizations should evaluate their 3–5-year technology roadmap and prioritize systems offering upgrade pathways or integration capabilities with emerging technologies. Some vendors provide backward compatibility, enhancing their adaptability over the years with new hardware and software capabilities.

#### 5.3.2. Expansion Phase (Years 3–4)

Based on validated results from the initial implementation, organizations can expand capabilities by adding specialized systems for high-priority applications or sports for which the initial technology demonstrated limitations. This might include the following:

1. Adding a laboratory-grade optical system for detailed research projects or specialized clinical assessments;
2. Implementing sport-specific solutions for unique environments (e.g., waterproof IMUs for aquatic sports);
3. Expanding the number of units to cover additional teams or athletes.

This phased approach ensures that investments are aligned with demonstrated needs and that staff have developed the necessary expertise to maximize the utility of more advanced systems.

Table 3 provides recommendations for primary and secondary technologies based on the specific focus of athletic organizations.

### 5.4. Addressing Common Implementation Challenges

When implementing MoCap systems across multiple sport organizations, several common challenges are often faced that must be proactively addressed to ensure successful adoption and sustained utility.

#### 5.4.1. Expertise Development

Many organizations lack the specialized biomechanics expertise necessary to maximize the value of MoCap systems. To address this challenge, the following steps are recommended:

1. Setting a budget for comprehensive staff training (typically 20–40 h per system) with regular refresher sessions;
2. Establishing clear standard operating procedures for data collection, processing, and interpretation;
3. Considering partnerships with academic institutions or consultants to supplement in-house expertise;
4. Developing simplified reporting templates that translate complex biomechanical data into actionable insights for coaches and athletes.

These strategies ensure that technical capabilities are matched with the expertise needed to derive meaningful insights [5].

#### 5.4.2. Data Integration

MoCap data often exists in isolation from other athlete monitoring systems, limiting its contextual value and practical utility. Organizations should carry out the following:

1. Prioritize systems that allow for integration (open APIs or established integration pathways) with current athlete management platforms;
2. Develop standardized data formats and naming conventions to facilitate cross-platform analysis;
3. Implement automated data processing workflows to reduce manual handling and potential errors;
4. Create visualization tools that combine MoCap metrics with other performance and health data.

Effective integration enhances the value of MoCap data by placing it within the broader context of athlete monitoring [62].

#### 5.4.3. Balancing Academic and Practical Needs

Athletic departments often struggle to balance rigorous scientific methodology with practical coaching needs. To address this tension, the following steps are recommended:

1. Establish clear use case priorities that distinguish between research projects and routine monitoring;
2. Develop separate protocols with appropriate methodological rigor for each purpose;
3. Create tiered reporting systems that provide immediate practical feedback for coaches while preserving detailed data for deeper analysis;
4. Schedule regular meetings between scientific staff and coaching teams to align objectives and expectations.

This balanced approach ensures that MoCap technology serves both scientific and practical purposes effectively [4].

#### 5.4.4. Ensuring Sustained Utilization

Many systems are purchased but underutilized after their initial implementation, particularly in large multi-sport organizations with high staff turnover. To promote sustained utilization, the following steps are recommended:

1. Develop long-term staffing and funding plans that extend beyond initial acquisition;
2. Establish fundamental testing protocols and standard operating procedures that can be systematically followed despite turnover;
3. Create a centralized knowledge repository documenting system capabilities, limitations, and best practices;
4. Implement regular system utilization reviews to identify and address barriers to adoption.

These strategies help ensure that investments in MoCap technology continue to provide value over their full lifecycle [16].

## 6. Future Directions and Research Gaps

Despite significant advances in MoCap technology, several critical research gaps remain that limit its optimal implementation in multi-sport settings. First, gender-specific validation studies are notably scarce, with most research conducted predominantly on male athletes. This gap is concerning given established biomechanical differences between genders that could affect system accuracy and calibration requirements [64,65]. Gender-based differences include variations in stride length, gait cycle duration, joint angles during movement, and landing mechanics, with females typically showing greater knee valgus angles and different propulsion patterns [64,65]. These fundamental movement pattern differences may require gender-specific calibration algorithms or validation protocols to ensure the optimal system accuracy across diverse populations. Future research should explicitly address this through balanced participant samples and stratified reporting of accuracy metrics [42].

Additionally, long-term reliability assessments are lacking across all technologies. Most validation studies focus on acute accuracy rather than system drift or reliability over extended periods (weeks to months), which is critical for longitudinal athlete monitoring. Research examining measurement stability across multiple sessions would better inform organizations about recalibration needs and data consistency expectations [24].

Furthermore, environmental transition capabilities remain underexplored. Few studies have examined how systems perform when athletes move between environments (from indoors to outdoors or from land to water), creating uncertainty for sports requiring such transitions. Standardized validation protocols for these scenarios would benefit triathlons, modern pentathlons, and similar multi-environment sports [45].

Lastly, the application of artificial intelligence for automated movement quality assessment represents a promising frontier. Current systems primarily track kinematics, but emerging research suggests AI algorithms could automatically identify technique flaws or injury risk patterns from motion data. Developing and validating such algorithms across diverse sporting movements would significantly enhance the practical utility of MoCap systems in high-performance settings [6].

## 7. Conclusions

Motion capture technology offers powerful tools for enhancing athletic performance and health, but implementing these systems requires a strategic, evidence-based approach. For athletic departments managing multiple sports, we recommend a phased implementation approach: first establishing a foundation with portable systems for high-priority teams, then expanding to specialized applications based on validated results. While this framework specifically addresses multi-sport organizational decision making, the evidence and implementation strategies presented are broadly applicable to any entity selecting MoCap technology, including independent coaches, single-sport programs, and research institutions.

Organizations should prioritize technologies with demonstrated validation in their specific sports, robust integration capabilities with existing systems, and appropriate accuracy for their primary objectives, whether in regards to performance optimization, injury prevention, or multi-sport flexibility. By carefully applying the cost–benefit principles outlined in this framework and focusing on translating complex biomechanical data into actionable insights, athletic departments can effectively leverage motion capture to enhance athlete performance, promote health, and maintain competitive advantage across diverse sporting disciplines.

## Figures and Tables

**Table 1:** Comparative analysis of motion capture technologies for sports applications.

Feature	Optical Marker-Based	IMU-Based	Markerless
**Accuracy**	High	Moderate	Low to moderate
**Sampling Rate**	High (100–1000+ Hz)	Moderate to high (60–500 Hz)	Moderate to high (30–250 Hz)
**Setup Time**	High	Low to moderate	Low to moderate
**Ecological Validity**	Low (lab environment, markers)	High (wearable)	Moderate to high (non-invasive)
**Capture Volume**	Limited by camera setup	Unlimited with signal amplifying towers (wireless range)	Limited by camera setup
**Environmental** **Flexibility**	Low (controlled lighting)	High (indoor/outdoor)	Moderate (lighting sensitive)
**Occlusion Handling**	Poor	Excellent	Moderate
**Cost Range**	USD 50–250 k+	USD 2–50 k	USD 0–100 k
**Expertise Required**	High	Low to moderate	Low to moderate
**Best Applications**	Detailed biomechanical research, clinical assessment	Field-based monitoring, workload assessment, aquatic sports	Team screening, technique analysis, tactical assessment
**Key Limitations**	Lab-bound; markers alter movement; influenced by soft tissue artifacts	Drift; magnetic interference; sensors are influenced by soft tissue artifacts	Variable accuracy, computational demands

Note: Accuracies of the biomechanical analyses from the MoCap data are evaluated and compared between each system with respect to their application in sports settings, number of cameras, and capture volume (environment), as well as the movement planes, which impact accuracy; accuracies in MoCap analyses are typically highest in the sagittal plane and lowest in the transverse during rotations. The setup time ratings are between-system comparisons; the required time for setup is highly dependent on the environment (i.e., if the systems are portable or permanently in one location), the specific system (e.g., multi- vs. single-camera or sensor, requiring wires or wireless), as well as the number and expertise of the staff carrying out the setup. Cost ranges are approximate and can vary significantly based on system specifications, software, and accessories. Accuracy descriptions are generalizations based on reviewed literature; specific system performance varies by task and implementation within sports environment.

**Table 2:** Comparative analysis of MoCap system performance across sporting environments.

Environment	System Type	Key Limitations	Best Applications
**Indoor Court**	Optical Marker-Based	Setup time (30–60 min) Limited volume Marker occlusion	Detailed biomechanical research Clinical assessment
	IMU-Based	Magnetic interference Position drift Requires calibration	Technique monitoring Load quantification
	Markerless	Variable accuracy Occlusion issues Computational demands	Team screening Technique analysis
**Aquatic**	Waterproof IMU	Magnetic interference Limited joint angles Signal attenuation	Stroke analysis Performance monitoring Workload assessment
	Underwater Camera	Refraction issues Limited volume Marker challenges	Technique analysis Qualitative assessment
**Outdoor Field**	GNSS-IMU	Environmental interference Stadium limitations Battery constraints	Team monitoring Workload assessment Tactical analysis
	Portable Markerless	Lighting sensitivity Background complexity Limited volume	Technique analysis Movement screening
	Fixed Markerless	Fixed installation Limited coverage Sport-specific training	Baseball pitching Golf swing analysis Specialized movements

Note: S = Sagittal plane, F = Frontal plane, T = Transverse plane. Accuracy values represent ranges from multiple validation studies and vary based on specific movements, joints, and implementation details.

**Table 3:** Implementation strategy based on operational constraints and workflow requirements.

Organizational Focus	Validated Technology Options	Application Context	Validation Evidence and Implementation Considerations
**Performance** **Optimization**	Portable IMU systems (Catapult Vector, STATSports Apex); Multi-camera markerless systems (Theia3D)	Field-based monitoring; Laboratory technique analysis	ICC > 0.80 for key metrics; RMSE 3.20–15.66° sagittal plane
**Injury Prevention and** **Rehabilitation**	Multi-sensor IMU systems (Xsens MVN); Mid-range optical systems (6–8 cameras)	Field assessments; Clinical baseline testing	Angular accuracy 2–8°; Comparable spatial accuracy to high-end systems
**Multi-Sport** **Flexibility**	Markerless systems (Theia3D); Team monitoring IMU systems (Xsens MVN, Kinexon)	Training facility deployment; Multi-team practice monitoring	ICC > 0.9 for temporal variables; Position RMSE 8–9 cm
**Indoor Court Sports**	Multi-camera markerless systems (e.g., Theia3D, DARI Motion)	IMU-based systems for detailed assessment	- Optimize camera positioning to minimize blind spots - Develop rapid calibration protocols - Address lighting and background challenges
**Outdoor Field Sports**	GNSS-integrated IMU systems with RTK capabilities	Portable markerless systems for technique zones	- Verify accuracy in actual usage environments - Develop strategies for signal interference - Create weather contingency protocols
**Aquatic Sports**	Waterproof IMU systems with specialized mounts	Underwater camera systems	- Position sensors away from metal infrastructure - Develop waterproofing maintenance protocols - Address wireless transmission limitations

Note: Technology selection should be based on validated performance metrics for specific applications rather than hierarchical preferences. All quantitative values represent independently validated accuracy metrics from peer-reviewed studies. Organizations should verify current their validation status as technology capabilities evolve rapidly.

## Data Availability

No new data was created in this study. Data sharing is not applicable to this article.

## Abbreviations

MoCap: Motion Capture; IMU: Inertial Measurement Unit; GNSS: Global Navigation Satellite System; RMSE: Root Mean Square Error; ICC: Intraclass Correlation Coefficient; RTK: Real-Time Kinematic; LPS: Local Positioning System; BOS: Base of Support; EMG: Electromyography; FTE: Full-Time Equivalent.
